# Infestation and Distribution of Ectoparasitic Insects on the White-Footed Indochinese Rat (*Rattus nitidus*) in Southwest China

**DOI:** 10.3390/vetsci12121171

**Published:** 2025-12-09

**Authors:** Ya-Nan Li, Xue-Jiao Zhu, Xian-Guo Guo, Tian-Guang Ren, Yong-Guang Jing, Lei Zhang, Ti-Jun Qian

**Affiliations:** 1 Institute of Pathogens and Vectors, Key Laboratory of Ectoparasite Systematics and Evolution of Yunnan Provincial Education Department, Yunnan Provincial Key Laboratory for Zoonosis Control and Prevention, Dali University, Dali 671000, China; 2 School of Government Administration, Baoshan University, Baoshan 678000, China

**Keywords:** flea, sucking louse, Siphonaptera, Phthiraptera, rodent, taxonomy, ecology

## Abstract

**Background:** Ectoparasitic insects (fleas and sucking lice) on rodents can serve as vectors of some zoonotic diseases. **Objective:** To investigate the insect infestation and distribution on white-footed Indochinese rats (*Rattus nitidus*) in southwest China, a focus of zoonotic diseases. **Methods:** Field investigations were conducted at 116 survey sites between 2000 and 2024. Insects were identified under a microscope, and a series of calculations was conducted. **Results:** From 836 *R. nitidus* rat hosts, 3322 insects were identified as 24 species, including eight vector species. Fleas had more species (22) and fewer individuals (539) than sucking lice (two species with 2783 individuals). Male and adult hosts had higher insect infestations than females and juveniles (*p* < 0.05), and insect infestations varied in different environments. The insect spatial coefficients were higher than the critical values for determining aggregated distribution. The association coefficient between fleas and lice was *V* = 0.07 (*p* < 0.05). **Conclusions:** *Rattus nitidus* is susceptible to insect infestation, with the coexistence of multiple vector species. Fleas have much higher species diversity than sucking lice. Insect infestations exhibit sex and age biases of hosts as well as environmental heterogeneity. Fleas and lice are of aggregated and mutually independent distribution on *R. nitidus*. Multiple vector species on *R. nitidus* would probably increase the potential risk of transmission and focus persistence of related zoonotic diseases in southwest China.

## Simple Summary

Rodents harbor two groups of ectoparasitic insects, fleas and sucking lice. Some insects can serve as vectors of zoonotic pathogens. The white-footed Indochinese rat (*Rattus nitidus*) is a common rodent species in southwest China, a focus of zoonotic diseases. To investigate the insect infestation and distribution on *R. nitidus*, field investigations were conducted at 116 survey sites in southwest China between 2000 and 2024, and insects were identified under a microscope. A series of calculations was conducted. *Rattus nitidus* rats are susceptible to insect infestation. From 836 rat hosts, 3322 insects were identified as 24 species with eight vector species. Fleas have much higher species diversity with 22 species than sucking lice with only two species. Male and adult hosts have higher insect infestations than females and juveniles. Insect infestations vary in different environments. Fleas and lice are of aggregated and mutually independent distribution on hosts. The coexistence of multiple vector species would probably increase the potential risk of disease transmission and focus persistence.

## 1. Introduction

Rodents are not only important agricultural and forestry pests but also infectious sources and reservoir hosts of many zoonotic pathogens such as *Yersinia pestis* (pathogen of plague, a violent infectious disease), *Rickettsia mooseri* (pathogen of murine typhus or endemic typhus), *Leptospira* spp. (pathogens of leptospirosis), and other pathogens of zoonotic diseases (zoonoses) [1,2]. Rodents often harbor two major groups of insects on their body surface: fleas (Order Siphonaptera) and sucking lice (Order Phthiraptera). As common ectoparasites, fleas and lice can cause annoyance to animals and humans through their stinging activity, and some fleas can directly parasitize animals or even humans, leading to tungiasis. Most importantly, fleas can serve as vectors of pathogens of some zoonotic diseases such as plague, murine typhus, flea-borne spotted fever, and bartonellosis. Through the stinging and bloodsucking activity of vector fleas, the pathogens of these diseases (*Y. pestis*, *R. mooseri*, *R. felis*, and *Bartonella* spp.) can be transmitted among rodent hosts, and even from rodents to humans [3,4,5,6,7,8,9,10,11,12,13]. Some fleas can act as intermediate hosts of tapeworms such as *Hymenolepis nana*, *Hymenolepis diminuta*, and *Dipylidium caninum*, which can cause the corresponding taeniasis of pets (cats, dogs, etc.), domestic animals, or even humans. Some studies have shown that fleas may be associated with pathogen transmission of tularemia, leptospirosis, rabbit myxomatosis, trench fever, feline leukemia, mycoplasmal disease, and Lyme disease [7,8,11]. Of sucking lice, the human louse (*Pediculus humanus*) is the confirmed vector of *Rickettsia prowazekii* (pathogen of epidemic typhus or louse-borne typhus), *Borrelia recurrentis* (pathogen of epidemic relapsing fever or louse-borne relapsing fever), and *Bartonella quintana* (pathogen of trench fever) [9,14,15,16]. Although rodent lice cannot directly transmit pathogens from rodents to humans, they can serve as reservoir hosts of some zoonotic pathogens such as *Y. pestis*, *Francisella tularensis*, and *R. mooseri* [16,17,18,19]. Southwest China is a natural focus of many zoonotic diseases, with the prevalence of plague, murine typhus, and bartonellosis within its territory [20,21,22,23,24,25,26,27,28,29,30]. Therefore, it is of medical and veterinary significance to study fleas and sucking lice on rodents in the region.

The white-footed Indochinese rat, or Himalayan field rat, *Rattus nitidus* (Hodgson, 1845), is a common rodent species in southwest China. Being a common pest species, *R. nitidus* frequently appears in residential areas and farmlands and often causes considerable damage to crops [31,32]. In addition, *R. nitidus* is also the infectious source and reservoir host of pathogens of some zoonotic diseases, such as plague, murine typhus, hemorrhagic fever with renal syndrome (HFRS), scrub typhus, leptospirosis, and other zoonotic diseases [33,34]. Previously, our research team once conducted a series of field investigations in southwest China and reported the infestation and distribution of ectoparasitic mites (chigger mites and gamasid mites) on rodents and other sympatric small mammals [35]. In the previous studies, we especially reported ectoparasitic gamasid mites on *R. nitidus* in Yunnan Province of southwest China [32]. Although we also collected fleas and sucking lice at some survey sites in the previous field investigations, our previous reports did not cover ectoparasitic insects (fleas and sucking lice), mainly because we did not have sufficient manpower and time to complete the taxonomic identification of these insects at that time. We have now completed the taxonomic identification of fleas and sucking lice collected from some rodent species, enabling us to conduct specialized research on these two groups of insects. Based on the result of insect taxonomic identification, the present study retrospectively reports the infestation and distribution of ectoparasitic insects (fleas and sucking lice) on the white-footed Indochinese rat (*R. nitidus*) in southwest China for the first time. The present study aims to provide scientific data on fleas and sucking lice on rodents, which is of medical and veterinary significance. The results of the present study will be conducive to the surveillance and control of vector insects and their associated zoonoses.

## 2. Materials and Methods

### 2.1. Field Collection and Taxonomic Identification

**Figure 1:**
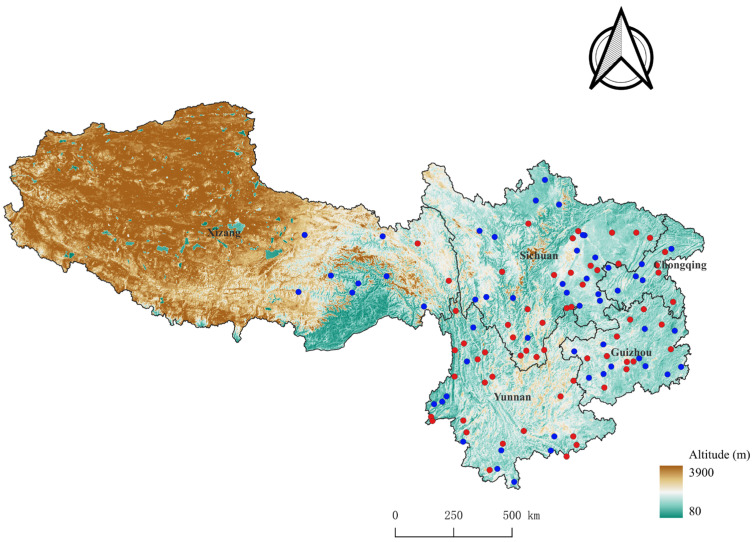
Field survey sites for ectoparasitic insects (fleas and sucking lice) on *Rattus nitidus* rats across the five provincial regions of southwest China (2000–2024). Annotation: The “●” represents the sites where *Rattus nitidus* rats were found (n = 61), and “●” stands for the sites without *R. nitidus* found (n = 55). The total survey sites were 116 sites (61 + 55 = 116).

The original data of the present study came from field investigations conducted at 116 survey sites across five provincial regions of southwest China (21°08′–33°41′ N, 97°21′–110°11′ E) between 2000 and 2024 (Figure 1). The 116 survey sites were those sites where both fleas and sucking lice were investigated, excluding the sites where the investigation involved only one group of insects (either fleas or lice) and the sites where the investigation involved only ectoparasitic mites (chigger mites or gamasid mites). The five provincial regions are Yunnan, Sichuan, Guizhou, Chongqing, and Xizang (Tibet). In Xizang Autonomous Region, the simultaneous investigations on both fleas and lice were only conducted at 10 survey sites in the eastern part (Figure 1). The vast majority of Xizang were not covered in the field investigations due to a series of reasons. Xizang Autonomous Region is a vast and sparsely populated territory, and the majority of the region is high-altitude plateaus with oxygen-thin air and relatively inconvenient transportation. Some high-altitude mountains are covered with snow all the year-round, making them unsuitable for field investigations. In addition, the lack of sufficient manpower and financial support also did not allow us to cover the entire territory of Xizang in the field surveys. In the field investigation at each survey site, mouse traps (18 × 12 × 9 cm; Guixi Mousetrap Apparatus Factory, Guixi, Jiangxi, China) were placed in different habitats to capture rodent hosts in the afternoon or evening. The investigated habitats included indoor habitats (human houses, stables, barns, and other resident areas) and outdoor habitats (farmlands, shrublands, woodlands, etc.). The captured rodent hosts were collected the next morning and transferred to a laboratory for insect collection. In a conventional way, each rodent host was separately placed in a large white tray to collect fleas and sucking lice on its body surface through combing and flipping the fur. All fleas and sucking lice collected from each rodent host were separately placed in a covered vial containing 70% (or 75%) ethanol for fixation and preservation. After the insect collection, each rodent host was identified to species according to its morphology, including the body size and shape, fur color, and a series of measurements such as body weight, body length, tail length, ear length, and hind foot length [36,37,38]. The flea and louse specimens collected were first digested with 5% or 10% sodium hydroxide (or potassium hydroxide), and then sequentially dehydrated using a gradient of 30%, 50%, 70%, 80%, 90%, 95%, and 100% ethanol. The dehydrated insects were placed in an anhydrous ethanol-xylene mixture (1:1 of volume ratio) and xylene for clarification, and finally mounted onto glass slides using Canadian balsam (or neutral balsam, or fir balsam) to make mounted slide specimens [13,39]. After drying mounted slide specimens in an oven, each insect (flea or louse) was carefully observed under a microscope (Olympus Company, Tokyo, Japan) for taxonomic identification. Based on taxonomic keys and morphological descriptions in relevant taxonomic books and literature, each insect was identified to species [13,40,41]. Representative specimens were deposited in the specimen repository of the Institute of Pathogens and Vectors in Dali University. The capture and use of rodent hosts for the research were officially approved by the local wildlife affairs authorities and the Animals’ Ethics Committee of Dali University.

### 2.2. Infestation Statistics

The constituent ratio (*C_r_*), prevalence (*P_M_*), mean abundance (*MA*), and mean intensity (*MI*) were calculated to reflect the infestation of *R. nitidus* with fleas and sucking lice. As an index of infestation frequency, the *P_M_* (%) refers to the percentage of infested rat hosts (*R. nitidus*) with ectoparasitic insects (fleas or sucking lice). The *MA* and *MI* are two indices reflecting infestation intensities. The *MA* refers to the average number of insects harbored by each examined rat host (insects/per examined host), and the *MI* refers to the average number of insects harbored by each infested rat host (insects/per infested host). The calculation formulas are as follows [42].

$$C_{r}=\frac{N_{i}}{N}\times 100\%;\;P_{M}=\frac{H_{i}}{H}\times 100\%;\;MA=\frac{N_{i}}{H};\;MI=\frac{N_{i}}{H_{i}}$$

In the above formulas, *N_i_* = the number of a certain flea or sucking louse species (species *i*) on a certain species of host (*R. nitidus* in the present study), *N* = the total number of all the flea or sucking louse species, *H* = the total number of hosts examined, and *H_i_* = the number of hosts infested with fleas or sucking lice.

Chi-square test (*χ*^2^) was used to test the statistical significance of *P_M_*, and the statistical significance of *MA* and *MI* was determined using the non-parametric. When *p* < 0.05, it is statistically significant; otherwise (*p* > 0.05), it is of no statistical significance.

### 2.3. Community Calculation

The community calculation involved the richness index (*S*), Shannon–Wiener diversity index (*H’*), Pielou evenness index (*E*), Simpson dominance index (*D*), and Berg–Parker index (*d*). The calculation formulas are as follows [35].

$$S=\sum S_{i};\;H^{\prime }=-\sum_{i=1}^{S}\left(\frac{N_{i}}{N}\right)\ln\left(\frac{N_{i}}{N}\right)\;E=\frac{H^{\prime }}{\ln S};\;D={\sum_{i=1}^{S}\left(\frac{N_{i}}{N}\right)}^{2};\;d=\frac{N_{\max}}{N}$$

In the above formulas, *S_i_* = species *i* (flea or louse) in the insect community, *N_i_* = the individuals of species *i*, *N* = total individuals of all species, ln = natural logarithm, and *N_max_* = the individuals of the most abundant dominant species.

### 2.4. Measurement of Spatial Distribution Patterns

Five types of spatial distribution coefficients were calculated to measure the spatial distribution patterns of ectoparasitic insects (fleas and sucking lice) among different individuals of the rat hosts, *R. nitidus*. The involved spatial distribution coefficients are the Dispersion coefficient (*C*), Clumping index (*I*), Patchiness index (m*/*m*), Cassie index (*C_A_*), and *K* index (*K*), and the formulas are as follows [43,44].

$$C=\frac{\sigma^{2}}{m};\;I=\frac{\sigma^{2}}{m}-1;\;m^{*}/m=\frac{m+\frac{\sigma^{2}}{m}-1}{m}\;C_{A}=\frac{\sigma^{2}-m}{m^{2}};\;K=\frac{m}{\frac{\sigma^{2}}{m}-1}$$

In the above formulas, *m* = mean of fleas and sucking lice on *R. nitidus*, *σ*^2^ = variance, m* = mean crowding.

### 2.5. Analysis of the Mutual Relationship Between Fleas and Sucking Lice

The association coefficient (*V*) was used to analyze the mutual relationship between fleas and sucking lice in host selection. The formula for calculating *V* is as follow [45,46].

$$V=\frac{ad-bc}{\sqrt{\left(a+b\right)\left(c+d\right)\left(a+c\right)\left(b+d\right)}}$$

In the above formula, *V *= the association coefficient between sucking lice (*X*) and fleas (*Y*); *a* = the number of hosts (*R. nitidus*) harboring both *X* (sucking lice) and *Y* (fleas); *b* = the number of hosts harboring only *Y* but not *X*; *c *= the number of hosts harboring only *X* but not *Y*; and *d *= the number of hosts with no *X* and *Y* at all. The values of *V* range from −1 to +1 [−1, 1]. The statistical significance of *V* is determined using the Chi-square test (*χ*^2^). When *V *> 0 and *p* < 0.05, it is of positive association between sucking lice (*X*) and fleas (*Y*); when *V *< 0 and *p* < 0.05, it is of negative association between *X* and *Y*; and when *V *= 0 or *V *≈ 0 and *p* < 0.05, it is of no association between *X* and *Y.*

### 2.6. Analysis of Interspecific Relationships

Spearman’s rank correlation coefficient (*r*) was used to analyze the interspecific relationships among different insect species (species of fleas and lice), and the results were visualized by a heat map. The correlation coefficient (*r*) ranges from −1 to 1 [−1, 1]. The Origin 2024 software was used to create a visual heat map of the interspecific relationships between any two species of fleas and sucking lice. The formula of Spearman’s rank correlation coefficient (*r*) is as follows [47]:

$$r=\frac{\sum_{i=1}^{n}\left(X_{i}-\bar{X}\right)\left(Y_{i}-\bar{Y}\right)}{\sqrt{\sum_{i=1}^{n}{\left(X_{i}-\bar{X}\right)}^{2}}\sqrt{\sum_{i=1}^{n}{\left(Y_{i}-\bar{Y}\right)}^{2}}}$$

In the above formula, *X_i_* is the *i*-th observation value of variables *X*, $\bar{X}$ is the mean value of variables *X*, *Y_i_* is the *i*-th observation value of variables *Y*, $\bar{Y}$ is the mean value of variables *Y*, and *n* represents the number of samples.

## 3. Results

### 3.1. Infestation and Distribution of Ectoparasitic Insects on R. nitidus

**Figure 2:**
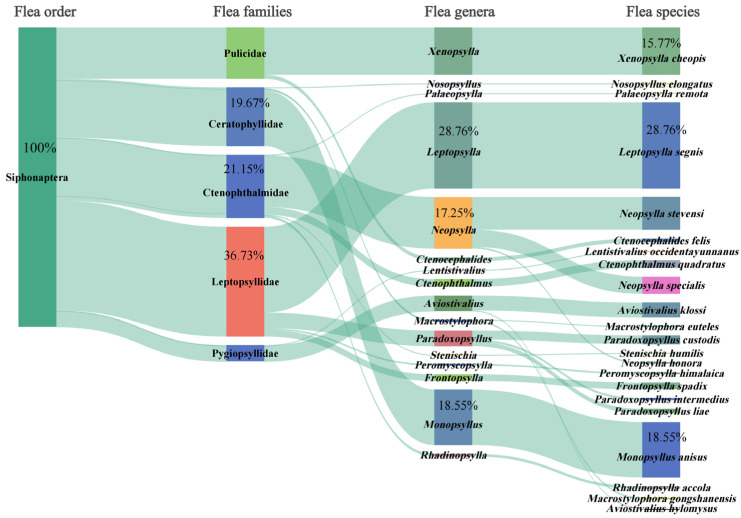
The visualization of constituent ratios (*C_r_*) of 539 fleas across different taxonomic levels (orders, families, genera, and species) on *Rattus nitidus* rats (hosts) in southwest China (2000–2024). Annotation: The shade width of each patch (band or string) represents the corresponding constituent ratio (*C_r_*) of fleas at a certain taxonomic level, order, family, genus, or species.

**Figure 3:**
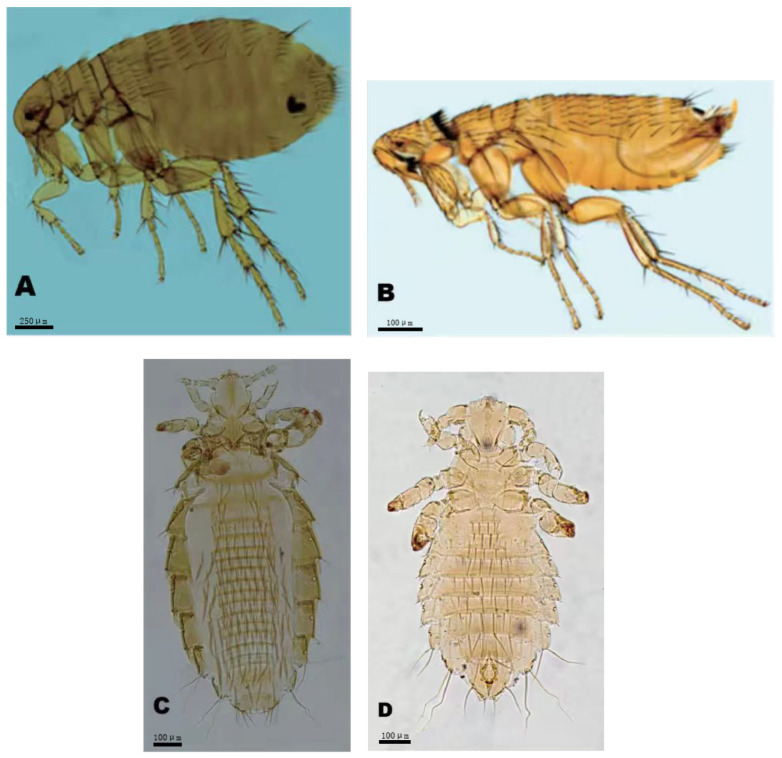
The photographs of four representative insect species identified from *Rattus nitidus* rats (hosts) in southwest China (2000–2024). Annotation: (**A**) The flea *Xenopsylla cheopis* (Rothschild, 1903) (♀, 10 × 20). (**B**) The flea *Leptopsylla segnis* (Schönherr, 1811) (♂, 10 × 20). (**C**) The sucking louse *Hoplopleura pacifica* Ewing, 1924 (♀, 10 × 20). (**D**) The sucking louse *Polyplax spinulosa* (Burmeister, 1839) (♂, 10 × 20).

Across the five provincial regions of southwest China, the field investigation for ectoparasitic insects (fleas and sucking lice) was conducted at 116 survey sites. A total of 836 white-footed Indochinese rats (*R. nitidus*) were captured from 61 of 116 survey sites (Figure 1). Among the 61 survey sites where *R. nitidus* rats were captured, there were 39 survey sites where *R. nitidus* rats were infested with ectoparasitic insects, including 15 sites where the rats were simultaneously infested with two groups of insects (fleas and lice), 6 sites where the rats were only infested with fleas, 18 sites where the rats were only infested with lice, totaling 39 sites (15 + 6 + 18 = 39). Of 836 *R. nitidus* rats captured, 355 rats were infested with ectoparasitic insects, and a total of 3322 ectoparasitic insects were collected from the rats. The 355 positive *R. nitidus* rats infested with ectoparasitic insects include 59 rats that were simultaneously infested with two groups of insects (fleas and lice), 100 rats that were only infested with fleas, and 196 rats that were only infested with lice, totaling 355 rats (59 + 100 + 196 = 355). The 3322 ectoparasitic insects were taxonomically identified as 7 families, 18 genera, and 24 species in two orders, Siphonaptera (flea) and Phthiraptera (louse). The identified 24 insect species include 22 flea species (Figure 2) and two sucking louse species (*Hoplopleura pacifica* and *Polyplax spinulosa*). The overall infestation indexes of ectoparasitic insects (fleas + lice) on the rat hosts were *P_M_* = 42.46%, *MA *= 3.97 insects/per examined host, and *MI *= 9.36 insects/per infested host, respectively. Of 24 insect species identified, eight flea species can serve as the vectors or potential vectors of pathogens of some zoonotic diseases such as plague, murine typhus, flea-borne spotted fever and bartonellosis. These vector flea species are *Xenopsylla cheopis*, *Leptopsylla segnis*, *Monopsyllus anisus*, *Ctenocephalides felis*, *Neopsylla specialis*, *Paradoxopsyllus custodis*, *Frontopsylla spadix*, and *Stenischia humilis*. Of these vector fleas, *Xenopsylla cheopis* is the most important vector of *Y. pestis* (pathogen of plague) and *R. mooseri* (pathogen of murine typhus) in southwest China. Table 1 lists the eight vector flea species and the zoonotic diseases they are associated with. Figure 3 shows the morphologies of two representative flea species (*X. cheopis* and *L. segnis*) and two sucking louse species (*H. pacifica* and *P. spinulosa*), which were taken under a microscope (10 × 20).

### 3.2. Infestation Comparison of Fleas and Sucking Lice on R. nitidus

A total of 3322 ectoparasitic insects collected from *R. nitidus* were identified as 24 species, 18 genera, seven families, and two orders. Of 3322 insects (24 species), 539 fleas were identified as 22 species, 16 genera, and five families in one order (Siphonaptera), and 2783 sucking lice belonged to two species, two genera, and two families in one order (Phthiraptera). The number of flea species accounted for 91.67% (22/24) of all insect species, and the number of flea individuals accounted for 16.23% (539/3322) of all insect individuals. The number of sucking louse species accounted for 8.33% (2/24) of all insect species, and the number of sucking louse individuals accounted for 83.77% (2783/3322) of all insect individuals. In comparison with sucking lice (two species with 2783 individuals), fleas exhibited high species richness (22 species) but low individual abundance (539 individuals). The overall infestation indexes of fleas on *R. nitidus* were *P_M_* = 19.02%, *MA *= 0.64, and *MI *= 3.39, and those of sucking lice were *P_M_* = 30.50%, *MA *= 3.33, and *MI *= 10.91, respectively. The overall infestation indexes of sucking lice were significantly higher than those of fleas (*p* < 0.05).

A total of 159 *R. nitidus* rats were infested with fleas, including 100 rats only infested with fleas, and 59 rats infested with both fleas and sucking lice. A Sankey diagram was used to visualize the hierarchical taxonomic structure of fleas (Siphonaptera) and the proportion of flea individuals at each taxonomic level. This diagram visually revealed the proportion distribution (constituent ratios, *C_r_*) of 539 fleas across the following different taxonomic levels: one order (Siphonaptera), five families, 16 genera, and 22 species (Figure 2). At the levels of five families and 16 genera, 36.73% and 28.76% of fleas came from the family Leptopsyllidae (*C_r_* = 36.73%, 198/539) and the genus *Leptopsylla* (*C_r_* = 28.76%, 155/539). At the species level (22 species), the dominant flea species were *L. segnis*, *M. anisus*, and *X. cheopis*, accounting for 63.08% of total flea individuals (Table 2, Figure 2). Among the three dominant flea species, the prevalence (*P_M_* = 6.22%) and mean abundance (*MA *= 0.19) of *L. segnis* were higher than those of other dominant flea species (*p* < 0.05). The mean intensity of *X. cheopis* (*MI *= 3.54) was higher than that of other dominant flea species, but without statistical significance (*p* > 0.05) (Table 2).

A total of 255 *R. nitidus* rats were infested with sucking lice, including 196 rats only infested with lice, and 59 rats infested with both lice and fleas. Of two species, two genera and two families of sucking lice, the dominant louse species *H. pacifica* in the genus *Hoplopleura* and the family Hoplopleuridae accounted for 73.91% (*C_r_* = 73.91%, 2057/2783) of the total two louse species, while *P. spinulosa* in the genus *Polyplax* and the family Polyplacidae accounted for 26.09% (*C_r_* = 26.09%, 726/2783) of the total lice. The infestation indices of *H. pacifica* (*P_M_* = 23.44%, *MA *= 2.46, *MI *= 10.49) were significantly higher than those of *P. spinulosa* (*P_M_* = 9.33%, *MA *= 0.87, *MI *= 9.31) (*p* < 0.001). In addition, the infestation indices of the two louse species were also higher than those of the three dominant flea species (Table 2).

### 3.3. Insect Infestations on Different Sexes and Ages of Hosts

**Figure 4:**
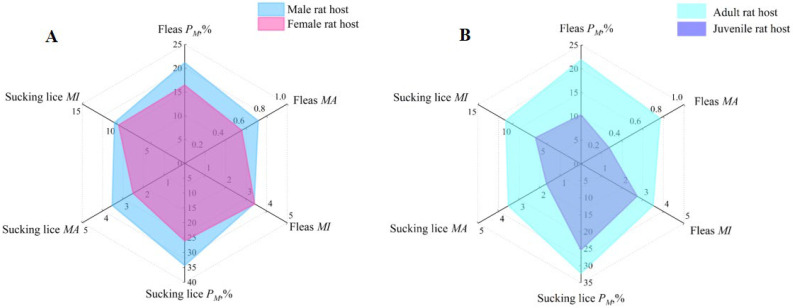
Radar chart visualization for insect infestations on different sexes and ages of rat hosts (*Rattus nitidus*) in southwest China (2000–2024). Annotation: (**A**) Insect infestations on different sexes of rat hosts (*R. nitidus*): the blue area represents the infestation indexes (*P_M_*, *MA*, and *MI*) of insects (fleas and sucking lice) on male rat hosts, and the pink one stands for the *P_M_*, *MA*, and *MI* of insects on female rat hosts. (**B**) Insect infestations on different ages of rat hosts (*R. nitidus*): the light blue area represents the infestation indexes (*P_M_*, *MA*, and *MI*) of insects on adult rat hosts, and the dark blue one stands for the *P_M_*, *MA*, and *MI* of insects on juvenile rat hosts.

A radar chart was used to visualize the infestation differences in insects (fleas and sucking lice) on different sexes and ages of rat hosts, *R. nitidus*. In the radar charts, the wider the areas, the higher the infestation indexes (*P_M_*, *MA*, and *MI*), and conversely, the narrower the areas, the lower the infestation indexes (Figure 4). The results showed that the infestation indices (*P_M_*, *MA*, and *MI*) of insects varied among different sexes and ages of rat hosts. Male rat hosts had higher *P_M_* and *MA* of sucking lice (*P_M_* = 34.40%, *MA* = 3.54) than female hosts (*P_M_* = 26.08%, *MA* = 2.52) with statistical significance (*p* < 0.05), but the louse *MI* difference on different sexes of rat hosts was of no statistical significance (*p* > 0.05). The differences in flea infestation indices on male and female hosts were of no statistical significance (*p* > 0.05). Adult rat hosts had higher *P_M_* and *MA* of fleas (*P_M_* = 21.90%, *MA* = 0.77) than juvenile hosts (*P_M_* = 10.33%, *MA* = 0.28) with statistical significance (*p* < 0.001), but the flea *MI* difference on different ages of rat hosts was of no statistical significance (*p* > 0.05). The differences in louse infestation indices on adult and juvenile rat hosts were of no statistical significance (*p* > 0.05) (Figure 4).

### 3.4. Fluctuations of Insect Infestation in Different Environments

**Figure 5:**
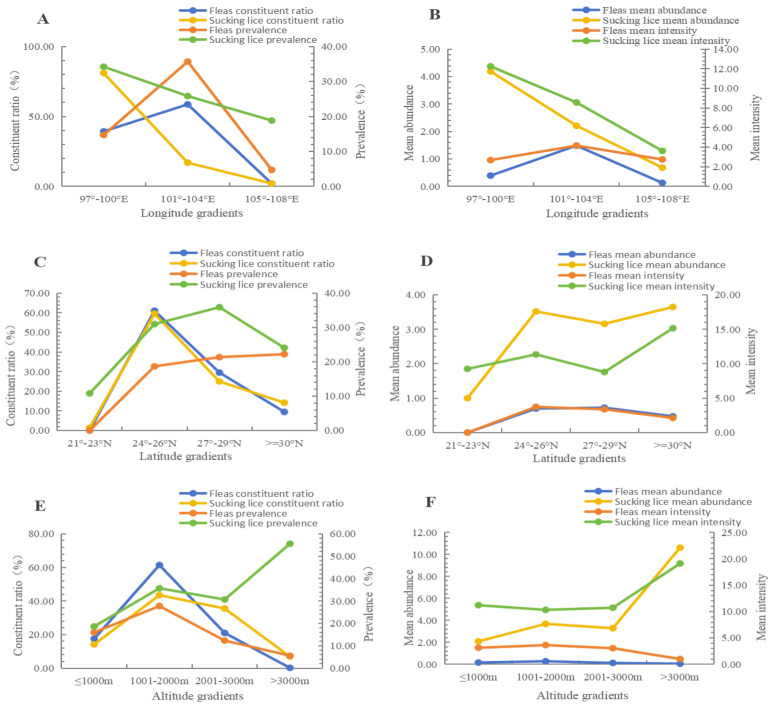
Infestation fluctuations of insects (fleas and sucking lice) on *Rattus nitidus* along different gradients of longitudes (**A**,**B**), latitudes (**C**,**D**), and altitudes (**E**,**F**) in southwest China (2000–2024).

The infestation indices of insects (fleas and sucking lice) on *R. nitidus* varied in different environments. Figure 5 shows the fluctuations of infestation indexes along different gradients of longitudes (A and B), latitudes (C and D), and altitudes (E and F). Table 3 revealed the variations in infestation indexes in different habitats and geographical landscapes. Along different longitude gradients, flea infestation indexes were highest at 101–104° E (*P_M_* = 35.68%, *MA* = 1.48, *MI* = 4.16), and louse infestation indexes were highest at 97–100° E (*P_M_* = 34.20%, *MA* = 4.19, *MI* = 12.25) with statistical significance (*p* < 0.05). Along different latitude gradients, fleas had the highest *P_M_* (22.22%) at ≥30° N and the highest *MA* (0.72) at 27–29° N (*p* < 0.05), and sucking lice had the highest *MA* at ≥30° N (3.65) and the highest *P_M_* at 27–29° N (35.91%) (*p* < 0.05). At different altitudes, fleas had the highest *P_M_* (27.74%) and *MA* (0.28) at 1001–2000 m (*p* < 0.001), and sucking lice had the highest *P_M_* (55.56%) and *MA* (10.61) at >3000 m (*p* < 0.001) (Figure 5). In different landscapes, the infestation indices of fleas in the mountainous landscape (*P_M_* = 27.47%, *MA* = 0.96, *MI* = 3.51) were higher than those in the flatland landscape (*P_M_* = 5.56%, *MA* = 0.10, *MI* = 1.89) (*p* < 0.05). The differences in flea infestation indices in different habitats were of no statistical significance (*p* > 0.05). The differences in louse infestation indices in different habitats and landscapes were of no statistical significance (*p* > 0.05) (Table 3).

### 3.5. Comparison of Community Indexes of Fleas and Sucking Lice

The flea community on *R. nitidus* had a low number of individuals (539) but a high number of species (22), while the louse community had a high number of individuals (2783) but a very low number of species (only two species). The number of species in the flea community was extremely higher than that in the louse community. The louse community had lower richness (*S*) and Shannon-Wiener’s diversity index (*H’*), but higher Pielou’s evenness index (*E*), Simpson’s dominance index (*D*), and Berg-Parker’s index (*d*) than the flea community (Table 4).

### 3.6. Spatial Distribution Patterns of Ectoparasitic Insects

The spatial distribution indexes of fleas (*C* = 6.00, *I* = 5.00, m*/*m* = 8.76, *C_A_* = 7.76, *K* = 0.13) and sucking lice (*C* = 67.92, *I* = 66.92, m*/*m* = 21.10, *C_A_* = 20.10, *K* = 0.05) on *R. nitidus* were all higher than the critical values of determining aggregated distribution (*C* > 1, *I* > 0, m*/m > 1, *C_A_* > 0, *K* > 0) (Table 5).

### 3.7. The Sex and Age Structure of Ectoparasitic Insects

**Figure 6:**
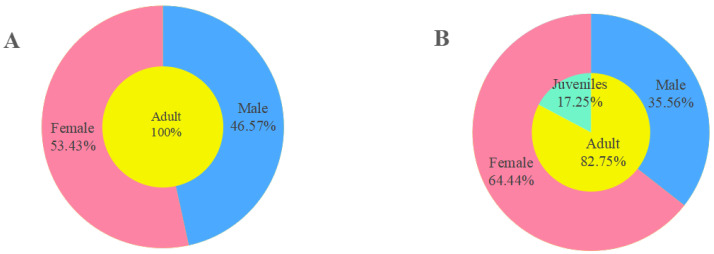
The sex and age structure of ectoparasitic insects (fleas and sucking lice) on *Rattus nitidus* in southwest China (2000–2024). Annotation: (**A**). The sex and age structure of fleas. (**B**). The sex and age structure of sucking lice.

Within the populations of fleas and sucking lice, the constituent ratios (*C_r_*) of female fleas (*C_r_* = 53.43%, 288/539) and female sucking lice (*C_r_* = 64.44%, 1484/2303) were higher than those of male fleas (*C_r_* = 46.57%, 251/539) and male lice (*C_r_* = 35.56%, 819/2303). The *C_r_* of adult lice (*C_r_* = 82.75%, 2303/2783) was much higher than that of juvenile lice (*C_r_* = 17.25%, 480/2783) (Figure 6). In the life cycle of fleas, only adults (male and female) are ectoparasites. Therefore, there were no juvenile fleas in the present study.

### 3.8. Mutual Relationship Between Fleas and Sucking Lice

Based on the contingency table of Table 6, the association coefficient (*V*) was used to measure the mutual relationship between fleas and sucking lice in host selection. The result showed that the association coefficient (*V*) was very close to zero (*V* = 0.07, *p* < 0.05) (Table 6).

### 3.9. Interspecific Relationships Among Different Insect Species

**Figure 7:**
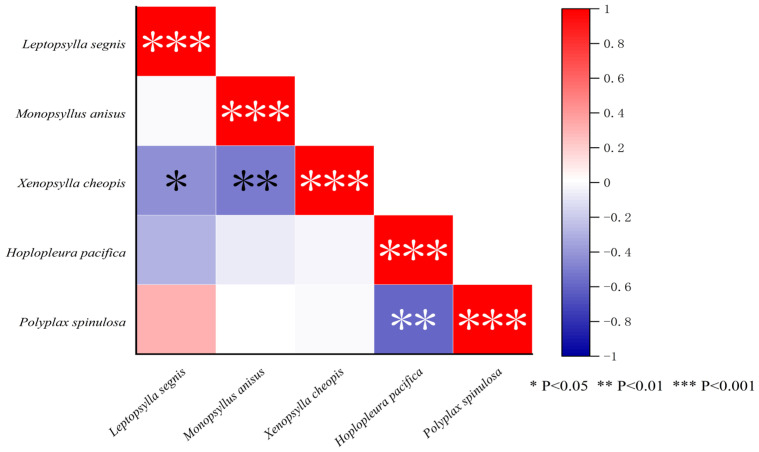
Heat map visualization for the interspecific relationship among the main species of fleas and sucking lice on *Rattus nitidus* in southwest China (2000–2024).

The interspecific relationships among the five main insect species (three flea species and two louse species) were analyzed using Spearman’s rank correlation coefficient (*r*), and the result was visualized using a heatmap. In the visualized figure, the red color represents a positive correlation (0 < *r* < 1), and the blue color represents a negative correlation (−1 < *r* < 0). The mark “***” indicates *p* < 0.001, “**” indicates *p* < 0.01, and “*” indicates *p* < 0.05. The results showed that there were varying degrees of positive or negative correlations between any two of the main insect species. The interspecific relationships between two louse species (*H. pacifica* and *P. spinulosa*) exhibited a negative correlation (*r* = −0.58, *p* < 0.01). The relationships between any two of the three dominant flea species also showed a negative correlation, and the negative correlation between *X. cheopis* and *M. anisus* was the most prominent, with *r* = −0.50 (*p* < 0.01). The correlation coefficient (*r*) between any two of the flea species and the louse species was very close to zero, and for example, *r* = −0.010 between the flea *X. cheopis* and the louse *P. spinulosa*, and *r* = 0.014 between the flea *M. anisus* and the louse *P. spinulosa* (Figure 7).

## 4. Discussion

The southwest China in the present study includes five provincial regions (Yunnan, Guizhou, Sichuan, Chongqing, and Xizang), and it is a very vast territory (2,341,467 km^2^) with a population of approximately 205 million. Southwest China is a natural focus of many zoonotic diseases, such as plague, murine typhus, bartonellosis, leptospirosis, hemorrhagic fever with renal syndrome (HFRS), scrub typhus, and others. Being two important flea-borne infectious diseases, plague and murine typhus were once prevalent in Yunnan, Sichuan, and Xizang, with a series of human cases reported successively [57,58,59,60,61,62]. Recent studies have shown that Yunnan and Sichuan are important foci of bartonellosis with rodents, cats, and pets serving as the main sources of infection [26,63]. A total of 1,157 human cases of murine typhus were once reported in Xishuangbanna Prefecture, Yunnan Province of southwest China in 2011, with an incidence of 102.10/100,000 [60]. Therefore, it is of medical and veterinary significance to study the vector of these diseases in the region.

In the present study, white-footed indochinese rats (*R. nitidus*) were found in 61 of 116 survey sites, and the 61 sites with the rats captured are distributed across all five provincial regions of southwest China (Figure 1). The result further supports the view that *R. nitidus* is a common and widely distributed rat species in southwest China [32,64,65]. The result of the present study showed that 24 species and 3322 individuals of ectoparasitic insects (fleas and sucking lice) were identified from *R*. *nitidus*, and the overall infestation indexes reached *P_M_* = 42.46%, *MA* = 3.97 insects/per examined host, and *MI* = 9.36 insects/per infested host. The result indicates that *R. nitidus* is susceptible to the infestation of fleas and sucking lice. Being a common rodent species in residential areas and farmlands, *R. nitidus* has a close relationship with human life and activities [31,32]. Besides causing damage to crops, *R. nitidus* is also the infectious source and reservoir host of some zoonotic pathogens such as *Y. pestis*, *R. mooseri*, *Leptospira* spp., and others [33,34]. The frequent presence of ectoparasitic insects (especially fleas) on rats *R. nitidus* facilitates the pathogen transmission among different rats and even from rats to humans.

Of 24 insect species identified, 22 were flea species, and only two were louse species, indicating that the species diversity of fleas is much higher than that of sucking lice. To date, there have been nearly 3000 flea species recorded in the world, and more than 600 flea species documented in China [66,67,68]. Of abundant flea species, some of them can be the effective vectors of pathogens of some zoonotic diseases such as plague, murine typhus, flea-borne spotted fever, and bartonellosis [3,5,6,7,8,9,10,11]. Of 22 flea species identified from *R. nitidus*, eight are vector species, which can effectively transmit *Y. pestis*, *R. mooseri*, *R. felis*, *Bartonella* spp., and some other zoonotic pathogens [5,7,8,9,11,13,48,49,50,51,52,53,54,55,56]. These vector species are *X. cheopis*, *L. segnis*, *M. anisus*, *C. felis, N. specialis*, *P. custodis*, *F. spadix*, and *S. humilis* (Table 1). In southwest China, there are two types of plague foci, the domestic rodent focus and the wild rodent focus. *Xenopsylla cheopis* is the major vector of plague in the domestic rodent focus, and it is also the major vector of murine typhus in the focus and nearby areas [5,7,9,11,13,48,49]. *Frontopsylla spadix* and *N. specialis* are the major vectors of plague in the wild rodent focus [54,55]. *Xenopsylla cheopis, L. segnis* and *C. felis* can serve as not only the vectors of pathogens of plague (*Y. pestis*), murine typhus (*R. mooseri*), flea-borne spotted fever (*R. felis*), and bartonellosis (*Bartonella spp.*), but also the intermediate hosts of the tapeworms *H. nana, H. diminuta*, and *D. caninum* [5,7,8,9,11,13,48,49,50,52]. The co-existence of multiple flea vector species on *R. nitidus* rats would increase the potential risk of zoonotic disease transmission and the focus persistence.

In comparison with sucking lice (two species with 2783 individuals), fleas exhibited high species richness (22 species), but low individual abundance (539 individuals) on the rat host *R. nitidus*. The overall infestation indexes of sucking lice (*P_M_* = 30.50%, *MA* = 3.33, and *MI* = 10.91) were significantly higher than those of fleas (*P_M_* = 19.02%, *MA* = 0.64, *MI* = 3.39) (*p* < 0.05). Although sucking lice exhibited higher infestation burdens (high infestation indexes) than fleas, the flea community showed much higher species richness (*S*) and Shannon-Wiener’s diversity index (*H*′) than the louse community (Table 4). The result reflects that the louse community is much simpler with much lower species diversity than the flea community. The simple structure of the community with low species diversity of sucking lice (only two species) is obviously associated with their specific life cycle process and high host specificity. Sucking lice are obligatory ectoparasites of eutherian mammals (Eutheria) with their entire life cycle (egg, nymph, and adult) permanently existing on their hosts. During a long history of co-evolution with hosts, sucking lice have developed high host specificity. The high host specificity of sucking lice means that one louse species only parasitizes very few species (even one or two species) of hosts, and one host species only harbors very few species (even one or two species) of lice [19,69,70,71]. The life cycle of fleas comprises four stages (eggs, larvae, pupae, and adults), and only adults (females and males) are ectoparasites of warm-blooded animals, birds (Aves), and mammals (Mammalia) [11,72,73,74,75,76]. This is why only male and female adults were collected from the body surface of *R. nitidus* rats in the present study. In comparison with sucking lice, many flea species exhibit low host specificity, meaning that one host species can harbor quite a few flea species and one flea species can parasitize a number of host species [7,11,74,77]. In this study, there were as many as 22 flea species found on the rat host *R. nitidus*, but only two sucking lice species were identified from the rat. The results further demonstrate the significant differences in host specificity between these two types of insects. The low host specificity allows fleas to transmit relevant zoonotic pathogens among different wild animals (especially rodents) and even from wild animals to humans [78,79]. Due to high host specificity, sucking lice have a much weaker potential for disease transmission among different animal hosts than fleas [80,81,82].

In the present study, the infestation indices (*P_M_* and *MA*) of ectoparasitic insects (fleas and sucking lice) were significantly higher on male and adult hosts (*R. nitidus*) than on female and juvenile hosts, indicating that the host *R. nitidus* has sex and age biases when it is infested with fleas and sucking lice (Figure 4). The sex and age biases of hosts are a common phenomenon in endoparasite infection and ectoparasite infestation. Some reports have shown that male and adult animal hosts have higher susceptibility to parasite infection than female and juvenile ones. The results of the present study are consistent with the previous reports [83,84]. The sex and age biases of hosts for parasitic infections involve a series of complex mechanisms. Because of competition for mating, male hosts (rodents and other animals) usually need to consume more energy than female ones, and this makes the males more vulnerable and susceptible to parasitic infections. The high level of testosterone in male hosts can also lower their immunity against parasitic infections [85,86]. In comparison with juvenile hosts, adult hosts have a large body surface area, which allows them to harbor more ectoparasites. Due to foraging needs, adult hosts have more activities than juvenile ones, and this makes the adults have more exposure to ectoparasite infestation than the juveniles [37]. The sex and age biases of hosts reflect the influence of host status (sexes and ages) on ectoparasitic infestation. Besides the influence of host status, the infestation of ectoparasites can be affected by a series of environmental factors. In the present study, the infestation indexes of fleas and sucking lice on *R. nitidus* varied in different longitudes, latitudes, altitudes, habitats, and geographical landscapes, which reflects the environmental heterogeneity of ectoparasitic infestations on the same host species, *R. nitidus* (Table 3, Figure 5). Some previous studies have shown that the same host species may exhibit environmental heterogeneity in ectoparasitic infestation under different environmental conditions, and the results of the present study are in accordance with the previous studies [87,88].

In ecological practices, there are a few ways to study spatial distribution patterns of insect populations, and spatial distribution indices (*C*, *I*, m*/*m*, *C_A_*, *K*) are simple ways to do so [43,44]. The spatial distribution indices of both fleas and sucking lice on *R. nitidus* were all higher than the critical values of determining aggregated distribution (*C* > 1, *I* > 0, m*/*m* > 1, *C_A_* > 0, *K* > 0) (Table 5), indicating that both fleas and sucking lice are of aggregated distribution among different hosts. The aggregated distribution further indicates that ectoparasitic insects (fleas and sucking lice) are unevenly distributed among different rat individuals (*R. nitidus*). Some rat hosts harbor very few or no insects on their body surface, while other hosts harbor quite a few insects, even forming insect clusters on their body surface. The aggregation is beneficial to intraspecific cooperation, survival competition, mating and reproduction, and population continuation of the parasites [87,89]. In addition, the aggregated distribution of some pests may also be related to their effective self-defense and survival protection mechanisms [90,91].

In the present study, the association coefficient (*V*) was used to measure the mutual relationship between fleas and sucking lice in host selection, and the association coefficient (*V*) was very close to zero (*V* = 0.07, *p* < 0.05) (Table 6). Spearman’s rank correlation coefficient (*r*) was used to analyze the interspecific relationships between different species of fleas and sucking lice. The results showed that the interspecific relationships between two louse species and between any two of the three dominant flea species all showed a negative correlation. The correlation coefficient (*r*) between flea species and louse species; however, was very close to zero (Figure 7). The nearly zero values of *V* between two insect groups (fleas and sucking lice) suggest that the infestations of two insect groups (fleas and sucking lice) on *R. nitidus* are mutually independent [45,46]. The infestation of fleas on *R. nitidus* does not significantly affect the infestation of sucking lice, and vice versa. The negative values of *r* between any two of fleas or sucking lice suggest that there may be interspecific competition among some species within the same insect group, fleas or sucking lice [92,93,94,95,96].

Within the populations of fleas and sucking lice, the constituent ratios (*C_r_*) of female fleas and sucking lice were higher than those of male ones. For most insects, female adults usually have longer lifespan than the males, and this can explain why fleas and sucking lice have more female individuals with high *C_r_* within their populations [97,98]. For the age structure of sucking lice, we cannot properly explain why the *C_r_* of adult lice was much higher than that of juvenile lice (Figure 6), and more research work remains to be performed in the future.

## 5. Conclusions

The white-footed Indochinese rat (*R. nitidus*) is susceptible to the infestation of fleas and sucking lice, and sucking lice have significantly higher infestation frequency and intensity than fleas. However, the community structure of fleas is much more complex with much higher species diversity than that of sucking lice. Multiple vector flea species co-exist on the rat host *R. nitidus*. The dominant flea species are *L. segnis*, *M. anisus* and *X. cheopis*, and the major louse species is *H. pacifica*. The infestations of both fleas and sucking lice exhibit the sex and age biases of hosts and environmental heterogeneity. Both fleas and sucking lice are of aggregated distribution among different individuals of *R. nitidus*. The infestations of fleas and sucking lice on *R. nitidus* are independent of each other, and there is almost no mutual association or correlation between these two groups of insects.

## Figures and Tables

**Table 1:** The eight vector flea species found on *Rattus nitidus* in southwest China and the zoonotic diseases they are associated with (2000–2024).

Vector Flea Species	No. of Fleas and Constituent Ratios (*C_r_*, %)		The Zoonotic Diseases the Fleas are Associated with	Supporting Literature Cited
	No.	*C_r_*, %		
*Xenopsylla cheopis*	85	15.77	plague, murine typhus, flea-borne spotted fever, bartonellosis, cestodiasis	[5,7,9,11,13,48,49]
*Leptopsylla segnis*	155	28.76	plague, murine typhus, flea-borne spotted fever, bartonellosis, cestodiasis	[5,11,13,48,49,50]
*Monopsyllus anisus*	100	18.55	plague, pseudotuberculosis, swine erysipelas, listeriosis	[13,48,51]
*Ctenocephalides felis*	7	1.30	plague, murine typhus, flea-borne spotted fever, bartonellosis, cestodiasis, feline leukemia, allergic dermatitis, helminthiasis	[5,7,8,9,11,13,49,52]
*Neopsylla specialis*	31	5.75	plague	[5,13,53,54]
*Paradoxopsyllus custodis*	16	2.97	plague	[13]
*Frontopsylla spadix*	12	2.23	plague	[13,53,54,55]
*Stenischia humilis*	1	0.19	plague	[13,56]
Total	407	75.51		

**Table 2:** Infestation indices of the main species of fleas and sucking lice on *Rattus nitidus* in southwest China (2000–2024).

Main Species of Fleas and Sucking Lice	No. of Hosts		No. and *C_r_* of Fleas and Sucking Lice		Infestation Indexes		
	Examined	Infested	No.	*C_r_*, %	*P_M_*, %	*MA*	*MI*
*Leptopsylla segnis*	836	52	155	28.76	6.22 *	0.19 *	2.98
*Monopsyllus anisus*	836	29	100	18.55	3.47 *	0.12 *	3.45
*Xenopsylla cheopis*	836	24	85	15.77	2.87 *	0.10 *	3.54
*Hoplopleura pacifica*	836	196	2057	73.91	23.44 **	2.46 **	10.49 **
*Polyplax spinulosa*	836	78	726	26.09	9.33 **	0.87 **	9.31 **

Annotation: *C_r_* = constituent ratio (%), *P_M_* = prevalence (%), *MA* = mean abundance (insects/per examined rat host), and *MI* = mean intensity (insects/per infested rat host). The asterisks of “*” and “**” represent *p* < 0.05 and *p* < 0.001, respectively.

**Table 3:** Infestation variations in insects (fleas and sucking lice) on *Rattus nitidus* in different habitats and landscapes in southwest China (2000–2024).

Ectoparasitic Insects	Habitats and Landscapes	No. of Hosts		No. and *C_r_* of Fleas and Sucking Lice		Infestation Indexes		
		Examined	Infested	No.	*C_r_*, %	*P_M_*, %	*MA*	*MI*
Fleas	Habitats							
	Indoor	84	24	71	13.17	28.57 *	0.85 *	2.96
	Outdoor	752	135	468	86.83	17.95 *	0.62 *	3.47
	Total	836	159	539	100.00	19.02	0.64	3.39
	Landscapes							
	Mountainous	495	136	477	93.35	27.47 **	0.96 **	3.51 *
	Flatland	324	18	34	6.65	5.56 **	0.10 **	1.89 *
	Total	819	154	511	100.00	18.80	0.62	3.32
Sucking lice	Habitats							
	Indoor	84	32	343	12.32	38.10	4.08	10.72
	Outdoor	752	223	2440	87.68	29.65	3.24	10.94
	Total	836	255	2783	100.00	30.50	3.33	10.91
	Landscapes							
	Mountainous	495	151	2073	76.24	30.51	4.19	13.73
	Flatland	324	100	646	23.76	30.86	1.99	6.46
	Total	819	251	2719	100.00	30.65	3.32	10.83

Annotation: *C_r_*, *P_M_*, *MA* and *MI*, * and **, the same as in Table 2.

**Table 4:** Community indexes of fleas and sucking lice on *Rattus nitidus* in southwest China (2000–2024).

Ectoparasitic Insects	No.	Community Indexes				
		*S*	*H’*	*E*	*D*	*d*
Fleas	539	22	2.17	0.70	0.16	0.29
Sucking lice	2783	2	0.58	0.83	0.61	0.74

**Table 5:** Distribution pattern indexes of fleas and sucking lice on *Rattus nitidus* in southwest China (2000–2024).

Ectoparasitic Insects	No.	Distribution Pattern Indexes				
		*C*	*I*	m*/*m*	*C_A_*	*K*
Fleas	539	6.00	5.00	8.76	7.76	0.13
Sucking lice	2783	67.92	66.92	21.10	20.10	0.05

Annotation: *C* = Dispersion coefficient, *I* = Clumping index, m*/m = Patchiness index, *C_A_* = Cassie index, *K* = Index of *K* value.

**Table 6:** The contingency table for analyzing the mutual relationship between fleas and sucking lice on *Rattus nitidus* in southwest China (2000–2024).

Occurrence Frequency of Fleas and Sucking Lice on *Rattus nitidus*		Sucking Lice (*X*)		Total
		+	−	
Fleas (*Y*)	+	59 (*a*)	100 (*b*)	159 (*a* + *b*)
	−	196 (*c*)	481 (*d*)	677 (*c* + *d*)
	Total	255 (*a* + *c*)	581 (*b* + *d*)	836 (*n*)
*V* and Chi-square test		*V* = 0.07, *χ*^2^ = 4.04, *p* < 0.05		

## Data Availability

The raw data supporting the conclusions of this article will be made available by the authors on request.
